# Childhood Maltreatment and Psychological Distress Among Adolescents and Young Adults Living with HIV in Zimbabwe

**DOI:** 10.1007/s40653-026-00861-y

**Published:** 2026-04-07

**Authors:** Edson Chipalo, Ikenna Obasi Odii

**Affiliations:** 1 School of Social Work, College of Allied Health Sciences, University of Cincinnati, Cincinnati, OH USA; 2 College of Nursing and Health Sciences, Metro State University, Saint Paul, MI USA

**Keywords:** Childhood maltreatment, Psychological distress, Adolescents and young adults, HIV, Zimbabwe

## Abstract

Childhood maltreatment is one of the global public health crises that contributes to increased risk of developing mental health problems for young people living with HIV in resource-constrained settings. This study examines the association between childhood maltreatment and psychological distress among adolescents and young adults living with HIV (AYALWHIV) in Zimbabwe. This national cross-sectional survey used data from the 2017 Zimbabwe Violence Against Children Survey (N = 249 AYALWHIV). Four logistic regression models were performed to determine significant associations between childhood maltreatment and psychological distress as measured by Kessler 6 scale during the past 30 days. The findings indicated that AYALWHIV who experienced cumulative childhood maltreatment (58.8%; AOR = 3.43 [CI = 1.83–6.41]), including emotional abuse (69.7%; AOR = 3.43, CI = 1.83–6.41) and sexual abuse (58.8%; AOR = 2.97 CI = 1.05–8.41) were associated with higher odds of reporting moderate-to-severe psychological distress during the past 30 days after adjusting for other factors. Therefore, this study underscores the need for comprehensive interventions that prioritize tailored anti-stigma education, HIV prevention support groups, community violence prevention strategies, expanded access to mental health counseling services, and promotion of acceptance and empathetic environments for AYALWHIV in resource-constrained settings. Furthermore, integrating mental health and violence prevention services into HIV care through targeted policy initiatives is critical for building coordinated and sustainable support systems in communities where AYALWHIV lives in Zimbabwe.

## Introduction

Child maltreatment is one of the global public health crises that not only results in immediate harmful consequences, but also significantly increases the risk of long-term physical, mental, and behavioral health problems such as chronic disease, substance use disorders, depression, anxiety and other forms of distress when experienced during childhood (Flor et al., 2025; Leeb, 2008; Massullo et al., 2023; Seppälä et al., 2024; Widom, 2013; World Health Organization [WHO], 2022). World Health Organization (WHO) characterizes childhood maltreatment as any form of abuse and neglect that occur to children under the age of 18 (World Health Organization [WHO], 2022). Childhood maltreatment has been frequently defined as *any act or omission by parents or other caregivers resulting in a potential threat of harm to a child* (Leeb, 2008). Childhood maltreatment is characterized by exposure to physical abuse, emotional abuse, sexual abuse, neglect, and commercial or other forms of exploitation that result in actual or potential harm to the child’s health, survival, development, or dignity, occurring within a context of a relationship of responsibility, trust, and power (World Health Organization [WHO], 2022). Neglect can manifest in both emotional or physical forms, characterized by caregiver’s inability to provide for a child’s essential needs for survival such as food, shelter, and medical care, and the absence of insufficient emotional support, affection, and supervision necessary for healthy development of a child. Similar to other other forms of maltreatment, neglect has been associated with poor physical and health outcomes that includes depression, anxiety, and other long-term health conditions (Norman et al., 2012).

Although child maltreatment is a concept that has been widely adopted to understand the nature of violence that occurs during childhood, there is no universal consensus on the definition (Herrenkohl, 2005). The conceptual definition of child maltreatment varies significantly, often shaped by different environment, social and cultural context (Herrenkohl, 2005; Massullo et al., 2023; McCoy & Keen, 2022; Seppälä et al., 2024). However, childhood maltreatment has been widely studied for its connection to wide range of adverse health and psychosocial outcomes, including risk of psychological distress (Flor et al., 2025; Herrenkohl, 2005). However, there remains an important need for a more comprehensive understanding of how child maltreatment contributes to increased risk of psychological distress among vulnerable young people living with HIV/AIDS in low income countries, especially in Sub-Saharan African Countries (Chipalo 2024a; Chipalo and Odii 2025b; Mohling et al. 2024; Molebatsi et al. 2024; World Health Organization [WHO], 2022).

Zimbabwe is one of the Sub-Saharan landlocked country located in Southern Africa region with an estimated population of approximately 16.3–17.3 million people as of 2023–2024, and it has a predominantly young population, with around 62% under the age of 25 (United Nations Population Fund Zimbabwe, 2024). Zimbabwe remains one of the top countries that continues to be hit with higher burdens of HIV. As of 2021, Zimbabwe’s HIV/AIDS prevalence rate was 11.6%, with an estimated 1.3 million people living with HIV. Among these vulnerable people living with HIV, approximately 72,100 were children aged 0–14 and 77,300 adolescents aged 10–19 in Zimbabwe (UNICEF, 2023). Although UNAIDS does not break down exact prevalence estimates of HIV for youth in Zimbabwe (15–24 years old), the prevalence estimates indicate that HIV continues to disproportionately affect youth with estimated prevalence of 3.7% for female and 2.6% for males, reflecting high burden of HIV (UNAIDS, 2024). Most recent studies have shown that living with HIV can expose many vulnerable adolescents and youths to maltreatment, poor health conditions, suicide risk and severe mental health issues, especially those living in impoverished conditions (Chipalo & Jeong, 2023; Doku et al., 2023).

Zimbabwe not only has highest prevalence of HIV, but also child maltreatment is highly prevalent. Recent studies have shown a higher prevalence of child maltreatment that includes physical, sexual, and emotional abuse, deprivation, and neglect which are linked to wide range of poor health outcomes in Zimbabwe (Andersson et al. 2008; Chipalo, 2024c; Chipalo and Jeong 2023; Chipalo and Odii 2025a; Patel et al. 2021; Spies et al. 2012). For instance, in 2018, Childline Zimbabwe reported higher rates of childhood maltreatment, particularly among those living with HIV. In the same year, Childline Zimbabwe received about 25,000 reports of child abuse cases. Out of these reported incidences of child abuse affecting children, 26% were exposed to sexual abuse, 20% were exposed to physical abuse, 17% exposed to emotional abuse, 17% were exposed to neglect and 20% were expose to any one or more forms of these child maltreatments (Zimbabwe Childline, 2018). Despite these alarming child maltreatment prevalences, many studies have not examined the extent to which child maltreatment either single type or cumulatively contributes to risk of psychological distress, especially for vulnerable adolescents and young adults living with HIV in Zimbabwe. However, multitude of studies have consistently demonstrated that child maltreatment has significant harmful effects, which may lead to poor mental health outcomes in both children and youth, including those living with HIV/AIDS (Badr et al. 2018; Chipalo, 2024d; Chipalo and Odii 2025a, 2025b; Goldberg and Short 2016; Holingue et al. 2020; Kisely et al. 2018; Young-Wolff et al. 2019; Zhou et al. 2019).

Psychological distress refers to experiencing mental or emotional difficulties, including anxiety, depressive symptoms, emotional pain, and stress (Fordjour et al., 2020). Psychological distress has an impairing ability that affects cognitive functioning and increases behavioral difficulties for persons living with HIV/AIDS (Adom et al., 2021; Chipalo & Odii, 2023; Okumu et al., 2021; Paul & Eckenrode, 2015; Viertiö et al., 2021). Exposure to child maltreatment can further complicate psychological distress due to stigma and discrimination that people living with HIV experience in their daily lives (Banati & Idele, 2021; Kimera et al., 2020; Kip et al., 2022; Meade et al., 2009; Spies et al., 2012). Numerous cross-sectional studies have shown that children exposed to various forms of child maltreatment, such as physical, emotional, and sexual abuse and/or neglect, may experience increased mental health issues such as anxiety, mood disorders, distress, and increased risk of substance use (Chapman et al., 2004; Doku et al., 2023; Evans et al., 2002; Kaplow & Widom, 2007; Paul & Eckenrode, 2015). However, these prior studies do not provide the extent to which maltreatment experiences relate to psychological distress, particularly for young people living with HIV/AIDS in Zimbabwe. On the other hand, the study that was conducted in Uganda involving adolescents and youth living with HIV/AIDS stated that exposure to physical abuse exacerbates poor mental health conditions by adding another layer of trauma and stress to already existing difficult encounters in limited resource settings (Okumu et al., 2021).

Prior studies have consistently documented the adverse psychological consequences associated with specific forms of child maltreatment such as physical, emotional and sexual abuse. Exposure to physical abuse, for example, has been linked to heightened symptoms of anxiety, depression, and post-traumatic stress disorder (PTSD). Among young people living with HIV, experiences of physical abuse may further complicate HIV management and intensify psychological distress, particularly due to persistent fear and trauma-related stress responses (Filiatreau et al., 2022). Similarly, sexual abuse has been strongly associated with poor mental health outcomes among adolescents and youth in Zimbabwe (Chipalo, 2023; Chipalo & Jeong, 2023; Chipalo & Odii, 2023). Evidence from studies of survivors of sexual violence indicates elevated levels of psychological distress, including broader emotional difficulties, depression, anxiety, and PTSD symptoms (Ramaswamy & Seshadri, 2020). Beyond sexual abuse long-term psychological effects, sexual abuse can also diminish self-esteem and negatively affect overall health functioning (Chipalo, 2023; Oberoi et al., 2020; Ramaswamy & Seshadri, 2020; Schou-Bredal et al., 2022).

Emotional abuse is defined as verbal assaults, humiliation, or demeaning behaviors intended to undermine a child’s sense of worth, well-being and self-esteem (Kaplow & Widom, 2007; Mwakanyamale & Mbao, 2021). Recent studies have shown that emotional abuse is associated with low self-esteem, anxiety, depression, and social isolation (Chilemba et al., 2014; Nazir et al., 2023). The cumulative impact of emotional abuse may erode coping mechanisms, disrupt healthy relationship formation, and weaken access to supportive social networks are particularly critical for individuals living with HIV/AIDS (Bhana et al., 2021).

Although multitude of studies have shown that exposure to child maltreatment is a risk factor for poor health involving physical, mental health and behavioral health outcomes (Chipalo, 2023; Oberoi et al., 2020; Ramaswamy & Seshadri, 2020; Schou-Bredal et al., 2022; Chilemba et al., 2014; Kaplow & Widom, 2007; Mwakanyamale & Mbao, 2021; Nazir et al., 2023). These studies have predominantly examined child maltreatment and HIV-related mental health outcomes as separate phenomena, rather than exploring their intersection. There remains a paucity of research directly examining the influence of child maltreatment on psychological distress among youth living with HIV/AIDS in many low-income countries (Filiatreau et al., 2022), particularly in Zimbabwe.

Therefore, the current study examined the association between childhood maltreatment and psychological distress among adolescents and young adults living with HIV (AYALWHIV) in Zimbabwe. Consistent with prior studies demonstrating strong associations between childhood maltreatment and other poor health outcomes in the general population (Banati & Idele, 2021; Chilemba et al., 2014; Kimera et al., 2020; Kip et al., 2022; Meade et al., 2009; Spies et al., 2012), we hypothesize that: (1) AYALWHIV who experienced cumulative childhood maltreatment will have higher levels of psychological distress, and (2) AYALWHIV who experienced any specific childhood maltreatment types such as emotional, physical, or sexual abuse will have higher levels of psychological distress in the past 30 days compared to those who were never exposed to any of these forms of maltreatments. Understanding this relationship is essential for developing targeted prevention and intervention strategies aimed at reducing both exposure to maltreatment and its long-term mental health conditions for vulnerable AYALWHIV in Zimbabwe.

## Methods

### Study Design and Sampling Procedures

The current study used the 2017 Zimbabwe Violence Against Children Survey (ZVACS). This nationally representative household cross-sectional survey targeted males and females aged 13 to 24 years old. ZVACS used a three-stage, cluster-randomized design. In the first selection stage, 1,000 female enumerated areas (EAs) and 118 male EAs were randomly selected out of 29,365 EAs with a probability proportional to the size of the EAs in terms of household presentation. An enumeration area (EA) is a geographical zone surveyed by one or more census enumerators (Kemp, 2008). In the second selection stage, the survey data collection involved teams that mapped and listed all the structures and households in each selected EA. The survey teams then input the total number of eligible households in the EA into a Microsoft Access program explicitly developed for ZVACS household selection. The program randomly selected 30 households in the EA to administer the survey. In stage three of selection, one eligible participant (female or male, depending on the EA) was randomly selected by a CSPro-built computer program from the list of all eligible participants aged 13 to 24 in each household (Zimbabwe Ministry of Health and Child Care, 2019). The study used a split-sample approach, following WHO guidelines, to protect participant confidentiality and ensure reliable prevalence estimates of abuse exposure among males and females (Zimbabwe Ministry of Health and Child Care, 2019).

### Inclusion Criteria

Participants were eligible if they (1) lived in a selected household in Zimbabwe and were between 13 and 24 years old at the time of the survey. This age range was selected to ensure participants could developmentally respond to survey questions while reducing potential recall bias about childhood experiences. (3) Participants needed to be fluent in English, Shona, or Ndebele to understand the survey items. Youth who could not participate due to cognitive or significant physical impairments affecting communication were excluded from the survey. Additionally, youth living in institutional settings (e.g., hospitals, prisons, nursing homes) were excluded from the survey because ZVACS was the household-based survey (Zimbabwe Ministry of Health and Child Care, 2019).

### Data Collection Procedures

Interviewers took thorough precautions to ensure maximum privacy during the time the survey was conducted. Interviews were conducted in safe, private locations, such as outdoors, in a public space without risk of interruption (e.g., a community area, school, or church), or in an appropriate place in the home or yard. Before beginning survey work in a new community, the team leaders sought guidance from community leaders to identify locations where interviews could be conducted that will provide maximum privacy and confidentiality of participants. Interviewers confirmed that participants, parents, and other household members were comfortable with the interview location before conducting the actual interview. If no private location was available, the interviewers rescheduled for another time while the survey team was still in the community. An interviewer of the same sex conducted the survey with the participants. Only the interviewer and participant were present during the interview; no one else, including the minor participants’ parents/guardians or other minors in the householdwere allowed to listen in. The length of participant interviews ranged from 20 to 60 min.

### Sample Size

After data collection, 803 male participants and 7,912 females completed the individual questionnaire. The overall response rates for males and females were 66% and 72%, respectively. The final sample size across male and female participants was 8,715. In the current study, we focused on participants who reported living with HIV, which reduced our sample to 249 (n=231 females, and n=males) adolescents and young adults living with HIV (AYALWHIV) in Zimbabwe (Zimbabwe Ministry of Health and Child Care, 2019).

### Dependent Variable

Psychological distress was assessed using the Kessler-6 (K-6), a non-specific psychological scale developed by Kessler and colleagues in 2001 (Kessler et al., 2002). The K-6 consists of six items that ask about symptoms of depression and anxiety experienced during the past 30 days. Participants indicate how frequently they have felt: (a) so sad that nothing could cheer them up, (b) that everything was effortless, (c) worthless about everything, (d) nervous, (e) hopeless, and (f) restless. Each item is rated on a five-point Likert scale, with scores ranging from 0 to 4 (0 = none of the time, 4 = all the time). The total score, which ranges from 0 to 24, is calculated by summing up the responses to these six items. The K-6 has demonstrated internal reliability and construct validity, with Cronbach’s alpha ranging from 0.80 to 0.89. The K-6 scale has been used across contexts, including low-income countries and especially Sub-Saharan Africa, where it has shown moderate psychometric properties (Juan et al., 2019; Prochaska et al., 2012). Since there is no recommended cut off score, we used cutoff points for determining psychological distress from the previous study (Prochaska et al., 2012). Participants with scores from 0 to 4 were categorized as having “no psychological distress,” while those scoring from 5 to 24 were considered to have “moderate/severe psychological distress” in the past 30 days.

### Independent Variables

The primary independent variable *childhood maltreatment*, which refers to any act of commission or omission by parents or other caregivers that results in a potential threat of harm to a child under 18 years old (Leeb, 2008). In this study, childhood maltreatment variables include exposure to physical, emotional, and sexual abuse, and cumulative childhood maltreatment (i.e., physical, emotional, or sexual abuse).

*Childhood emotional abuse.* Participants were asked whether, as children, they were told by a caregiver that (1) they were not loved or did not deserve to be loved, the caregiver wished they had never been born or were dead, and (3) they were ridiculed or put down, such as being called useless or stupid, before the age of 18. Response options were never, once, a few times, many times, and don’t know/declined. These responses were dichotomized as “no” (never experienced any emotional abuse) and “yes” (never experienced any emotional abuse at least once, a few times, or many times).

*Childhood physical abuse* was assessed by asking participants whether they had ever experienced any of the following: (1) being punched, kicked, whipped, or beaten with an object; (2) being choked, smothered, attempted drowning, or intentionally burned; or (3) threats with a knife or other weapons before the age of 18. The response options were yes, no, and don’t know/declined. These questions were asked for each type of perpetrator: (1) intimate partner, peers, (3) parents, adult caregivers, adult relatives, and and other adults in the neighborhood. Responses were dichotomized as “no” (never experienced any physical abuse) and “yes” (experienced any physical abuse).

*Childhood sexual abuse.* Participants were asked whether they had ever: (1) received food, favors, or gifts in exchange for sex, participated in a sex video or photo, or been shown their sexual body parts in front of a webcam, (3) experienced unwanted sexual touching, including fondling, pinching, grabbing, or touching around their sexual body parts, experienced unwanted sexual attempts where the perpetrator tried to make them have sex against their will but did not succeed, (5) been physically forced to have sex, and (6) been pressured to have sex through harassment, threats, or deception before the age of 18. Responses were coded as “yes,” “no,” and “don’t know/declined.” These responses were further dichotomized as “no” (never experienced sexual abuse) and “yes” (experienced any type of sexual abuse).

*Cumulative childhood maltreatment*. A summative scale of childhood maltreatment experiences, including emotional, physical, and sexual abuse, was coded as a continuous variable ranging from 0 to 3. These scores were summed to create a total score, which was then dichotomized as “no” (never experienced any childhood maltreatment) or “yes” (experienced any childhood maltreatment).

#### Control Variables

Control variables included socio-demographic characteristics such as sex, age, highest level of education completed, marital status, and number of close friends, which were all assessed as categorical or nominal variables in the current study.

### Data Analysis

All statistical analyses were performed using SPSS version 29.0, with a significance level set at *p* <.05. Prior to analysis, diagnostic tests were conducted. Outliers were assessed using standardized residuals and Cook’s distance, and no influential outliers were identified. Multicollinearity among independent variables was checked using variance inflation factors (VIF) and tolerance statistics. All VIF values were below the recommended threshold (VIF < 5), indicating no significant multicollinearity in the current study

At the univariate level, descriptive statistics were computed using frequencies and percentages for categorical variables. Continuous variables were examined for normality using skewness and kurtosis statistics, and the Shapiro–Wilk test. Although psychological distress (K6) scores were assessed for distributional properties, the outcome variable was dichotomized; therefore, normality was not required for regression analyses. The choice between parametric and nonparametric techniques was guided by the level of measurement and the distributions of the variables. Because most variables were categorical and the dependent variable was binary, non-parametric tests (chi-square) and logistic regression were appropriateand met the analytical assumptions. At the bivariate level, chi-square tests were conducted to examine associations between independent variables and psychological distress, and cross-tabulations were used to obtain prevalence estimates between independent variables (child maltreatment types) and the dependent variable (psychological distress in the past 30 days)

At the multivariate level, four logistic regression models (unadjusted and adjusted) were estimated. Logistic regression was selected because the outcome variable was binary and did not require normally distributed predictors. Specifically, Model 1 examined the association between individual childhood maltreatment types and psychological distress in the past 30 days (unadjusted). In Model 2, we examined the association between individual childhood maltreatment types and psychological distress in the past 30 days while adjusting for sociodemographic and household characteristics. In Model 3, we assessed the association between exposure to cumulative childhood maltreatment and psychological distress in the past 30 days (unadjusted). In Model 4, we assessed the association between exposure to cumulative childhood maltreatment and psychological distress in the past 30 days while adjusting for sociodemographic factors.

### Ethical Considerations

The Zimbabwe, 2017 VACS followed World Health Organization (WHO) guidelines on ethics and safety for child abuse studies. The Medical Research Council of Zimbabwe, the Research Council of Zimbabwe, and the CDC Institutional Review Board independently reviewed and approved the survey protocol to ensure the protection of participants’ rights and welfare. The United States CDC and the Zimbabwe Ministry of Health and Childcare approved the survey protocol and informed consent documents (Zimbabwe Ministry of Health and Child Care, 2019a). Permission to use the VACS was granted by the CDC on April 28, 2023, upon request by the first author(s). Since this study used secondary data that is publicly available upon request, the University of Cincinnati Institutional Review Board was exempted from full review.

## Results

### Descriptive Characteristics of AYALWHIV

The sociodemographic characteristics of adolescents and young adults living with HIV (AYALWHIV) are presented in Table 1. Among the 249 AYALWHIV, 92.8% were females, 86.7% were between 18 and 24 years old, 60.2% had completed at least secondary education, 54.6% were married, and 69.5% had one or more close friends. Approximately 32.1% of AYALWHIV had experienced any form of childhood maltreatment at some point in their lives. However, the most highly prevalent types of child maltreatment reported included physical abuse (19.7%), followed by emotional abuse (13.3%), and sexual abuse (9.6%), respectively. Approximately 42.4% of AYALWHIV reported moderate-to-severe psychological distress in the past 30 days.

**Table 1:** Sample Characteristics of AYALWHIV (*N* = 249)

Variables	Frequencies	Percentages
**Psychological distress**,** past 30 days**		
No	143	57.4
Yes	106	42.6
**Demographic characteristics**		
Gender		
Males	18	7.2
Females	231	92.8
Age (years)		
13–17	33	13.3
18–24	216	86.7
Highest education level		
Primary or less than primary	58	23.3
Secondary or higher than secondary	150	60.2
Missing	41	16.5
Marital status		
Never married	113	45.4
Married	136	54.6
Number of close friends		
None	76	30.5
One or more	173	69.5
**Childhood maltreatment types**		
Childhood emotional abuse		
No	212	85.1
Yes	33	13.3
Missing	4	
Childhood physical abuse		
No	200	80.3
Yes	49	19.7
Childhood sexual abuse		
No	225	90.4
Yes	24	9.6
Cumulative childhood maltreatment		
No	169	67.9
Yes	80	32.1

Psychological distress was assessed using Kessler-6 during the past 30 days

### The Prevalence of Psychological Distress Across Childhood Maltreatment Among AYALWHIV

Table 2 displays the prevalence estimates of psychological distress among AYALWHIV based on different types of childhood maltreatment. The results show that AYALWHIV who experienced any form of childhood maltreatment were significantly associated with reporting moderate-to-severe psychological distress in the past 30 days (58.8%; *p*<.001). Specifically, emotional abuse (69.7%; *p* <.001) and sexual abuse (58.8%; *p* =.003) were both significantly associated with reporting moderate-to-severe psychological distress in the past 30 days. However, experiencing any physical abuse was not significantly associated with reporting psychological distress in the past 30 days among AYALWHIV(49.0%; *p* =.311).

**Table 2:** The Prevalence of Childhood Maltreatment and Psychological Distress Among AYALWHIV (*N* = 249)

Variables	Psychological Distress		
	No	Yes	*P*-value
	N (%)	N (%)	
Childhood physical abuse			0.311
No	118 (59.0)	82 (41.0)	
Yes	25 (51.0)	24 (49.0)	
Childhood emotional abuse			**< 0.001**
No	132 (62.3)	80 (37.7)	
Yes	10 (30.3)	23 (69.7)	
Childhood sexual abuse			**0.003**
No	136 (60.4)	89 (39.6)	
Yes	7 (29.2)	17 (70.8)	
Cumulative childhood maltreatment			**< 0.001**
No	110 (65.1)	59 (34.9)	
Yes	33 (41.3)	47 (58.8)	

Psychological distress was assessed using Kessler-6 in the past 30 days

### Association Between Experiencing Childhood Maltreatment and Psychological Distress Among AYALWHIV

The results of logistic regression are shown in Table 3. The results indicated that AYALWHIV who experienced any form of childhood maltreatment (cumulative) were more likely to report moderate-to-severe psychological distress in the past 30 days compared to those who never experienced them. This association was significant in both unadjusted and adjusted models (OR = 2.66 [CI = 1.54–4.58], AOR = 3.43 [CI = 1.83–6.41]). Similarly, AYALWHIV who experienced any emotional abuse (OR = 3.80 [CI = 1.72–8.39], AOR = 4.68 [CI = 1.75–12.55]) and sexual abuse (OR = 3.71 [CI = 1.47–9.31], AOR = 2.97 [CI = 1.05–8.41]) during childhood were significantly associated with higher odds of reporting moderate-to-severe psychological distress in the past 30 days in both unadjusted and adjusted models. In contrast, experiencing any physical abuse was not significantly associated with higher odds of reporting psychological distress in the past 30 days among AYALWHIV (OR = 1.38 [CI = 0.73–2.59], AOR = 1.25 [CI = 0.58–2.72]).

**Table 3:** Association between Childhood Maltreatment and Psychological Distress Among AYALWHIV

Variables	Psychological Distress	
	OR (95% CI)	AOR (95% CI)
Childhood maltreatment types	Model 1	Model 2
*Childhood physical abuse*		
* No*	Ref	Ref
* Yes*	1.38 (0.73–2.59)	1.25 (0.58–2.72)
*Childhood emotional abuse*		
* No*	Ref	Ref
* Yes*	3.80 (1.72–8.39) ***	4.68 (1.75–12.55) **
*Childhood sexual abuse*		
* No*	Ref (1.00)	Ref(1.00)
* Yes*	3.71 (1.47–9.31) **	2.97 (1.05–8.41) *
*Cumulative childhood maltreatment*	**Model 3**	**Model 4**
* No*	Ref	Ref
* Yes*	2.66 (1.54–4.58) ***	3.43 (1.83–6.41) ***

*= *p*<.05, **= *p*<.01, ***= *p*<.001, OR = Odds Ratios, AOR= adjusted odds ratios. Any childhood abuse = includes the combined emotional, physical, or sexual abuse. Models 1 and 3 = unadjusted models involving assessing bivariate associations only, Models 2 and 4 = adjusted for demographic characteristics (i.e., sex, age, education, marital status, and number of close friends

## Discussion

The current study examined the association between childhood maltreatment and psychological distress among adolescents and young adults living with HIV (AYALWHIV) in Zimbabwe. This study makes significant contribution by providing comprehensive and valuable insights into how a history of childhood maltreatment can pose serious risk for developing psychological distress among vulnerable AYALWHIV. Particularly, 42.6% of AYALWHIV reported some form of moderate-to-severe psychological distress, 70.8% had experienced sexual abuse, 69.7% emotional abuse, and 49% physical abuse, with an overall exposure rate of 58.8% to any type of childhood maltreatment in the current study. Previous studies have established a strong connection between exposure to trauma or maltreatment and the development of serious mental health conditions (Chipalo, 2023, 2024b, Chipalo, 2024c; Chipalo & Jeong 2023; Chipalo and Odii 2023), and these may be more prevalent among vulnerable individuals living with HIV in low-resource settings (Yang et al., 2018).

Childhood maltreatment has oftentimes been linked to an increased risk of developing mental disorders for vulnerable individuals living with HIV(Scott et al., 2023). Similar to other previous studies (Badr et al., 2018; Chipalo & Odii, 2023; Kattimani & Bharadwaj, 2020; Singh & Singh, 2020; Su et al., 2022; Wang & Chen, 2023), our current study revealed that AYALWHIV who experienced cumulative childhood maltreatment (i.e., physical, emotional, and/or sexual abuse) had higher level of moderate-to-severe psychological distress. This implies that cumulative experiences of maltreatment, particularly during a critical phase of childhood and adolescence development, can lead to significant life challenges, ongoing stressors, and psychological distress that may persist into later adulthood, especially for individuals living with HIV(Richter et al., 2014). Since childhood maltreatment often involves multiple forms of abuse, such as sexual, physical, emotional, or neglect, the cumulative effect of these experiences can intensify psychological distress in AYALWHIV compared to those exposed to only one type of childhood maltreatment. While many vulnerable individuals living with HIV encounter stigma, discrimination and other forms of social exclusion, exposure to maltreatment further complicates other psychosocial stressors. This implies that the higher the exposure to childhood maltreatment, the higher the levels of vulnerability to psychological distress among AYALWHIV. Therefore, this implies that cumulative exposure to child maltreatment is a risk factor for psychological distress requiring rigorus prevention mechanisms, which may be crucial for reducing psychological distress and improving health outcomes for AYALWHIV living in low-resource settings in Zimbabwe.

One of the most severe forms of child maltreatment is emotional abuse, which is widely known as a potential risk factor for decreased low self-esteem, increased isolation and psychological distress (Bhana et al., 2021; Chilemba et al., 2014; Kaplow & Widom, 2007; Mwakanyamale & Mbao, 2021; Nazir et al., 2023). Oftentimes, individuals living with HIV are subjected to discrimination, and stigma resulting in various elements of psychological distress, including increased anxiety, depression, feelings of worthlessness, isolation, shame, and diminished sense of belonginess. These prevailing persistent emotional traumas can also undermine self-esteem and make it extremely challenging for AYALWHIV to adopt positive coping strategies to overcome psychological distress (Chipalo & Odii; Oluwasola, 2014; Remien et al., 2019). Emotional abuse can be expressed as a form of internalized stigma, making individuals more susceptible to negative self-perceptions about themselves and risk of developing severe mental health problems (Chilemba et al., 2014; Kaplow & Widom, 2007; Remien et al., 2019). Specifically for individuals living with HIV, persistent emotional strain can further interfere with adherence to HIV treatment, and complicate involvement in health management (Filiatreau et al., 2022). Therefore, the consequences stemming from emotional abuse may act as a barrier to the development of healthy coping mechanisms, and thus perpetuating a cycle of psychological distress for vulnerable AYALWHIV in low resource settings.

Similar to other forms of child maltreatment, sexual abuse is a potent risk factor for numerous physical and mental health conditions that can last for a lifetime (Chipalo, 2024c; Chipalo and Odii 2023, 2025b; Hailes et al. 2019; Kisely et al. 2018). Although previous studies have not directly focused on individuals living with HIV(Ani, 2024), the current study revealed that AYALWHIV who experienced sexual abuse were at greater risk of developing moderate-to-severe psychological distress, supporting our hypothesized direction. Sexual abuse can greatly affect psychological well-being, resulting in more complex, if not compromised, trauma, shame, isolation, and elevated vulnerability to psychological distress such as severe anxiety, depression, posttraumatic stress disorder (PTSD) among AYALWHIV (James et al., 2022). Unlike other forms of child maltreatment that are perpetuated, sexual abuse can be dangerous because it also increases the risk of exposure to sexual transmitted diseases, and these may be attached with either directly or indirectly physical and mental health consequences for the victims. Thus, living with HIV can create multifaceted traumatic experiences, including stigma linked to both HIV and sexual abuse may prevent AYALWHIV from seeking important medical and psychological support, which can harm their treatment adherence and their ability to overcome prevailing psychological distress.

Finally, although cumulative exposure to child maltreatment increases the risk of developing psychological distress, when separated by individual types of child maltreatment, experiencing any form of physical abuse during childhood did not emerge as a significant predictor of psychological distress among AYALWHIV. Our findings contradict previous studies that identified exposure to physical abuse as a significant risk factor contributing to a wide range of mental health problems, trauma, and stress among AYALWHIV (Filiatreau et al., 2022; Okumu et al., 2021). Some of the potential possible indicators leading to this unexpected outcome may be viewed in terms of environmental, cultural, social and support systems available to AYALWHIV. In terms of cultural perspective, physical abuse may be perceived as part of disciplinary measures, and this is much common including in countries like Zimbabwe (Chipalo, 2023; Hlavka, 2014). Normalization and acceptance of physical abuse can obscure the recognition of psychological distress symptoms, even in the face of stigma and discrimination experienced by AYALWHIV. The presence of strong social support systems at both individual and community levels, can contribute to increased resilience and can serve as buffer against psychological distress (Meng et al., 2018), particularly for AYALWHIV in low-resource settings. Resilience can be characterized by supportive caregivers, peer networks, and community-level of support e.g., access to services. Regardless, there is need to increase development of intervention strategies to prevent all forms of physical abuse, even though this study has revealed that it may not be risk factor to development of psychological distress for AYALWHIV.

### Implications for Practice and Policy

The current study has provided robust evidenced by indicating that exposure to childhood maltreatment (i.e., sexual and emotional abuse) increases the risk of psychological distress among AYALWHIV. In order to mitigate the psychological distress experienced by AYALWHIV who experienced emotional abuse, it is important to implement community and public health interventions that can primarily focus on anti-stigma education programs, creating support groups designed explicitly for AYALWHIV, increasing access to mental health counseling services, and creation of supportive, empathetic community environments. Increasing access to HIV treatment and prevention, alongside enhancing public understanding of these challenging issues, can help reduce stigma and improve overall mental wellbeing for AYALWHIV. Mental health interventions should incorporate trauma-informed care into HIV treatment services, ensuring that mental health support and counseling are both accessible and tailored to the unique needs of AYALWHIV. Creating safe spaces should be established where survivors of sexual violence can share their experiences without fear of stigma or judgment in their communities. Investment in educational programs in schools and community agencies are needed to continue raising awareness, especially the health risks associated with sexual abuse and HIV-related stigma is important for building a more supportive and informed community in low resource settings in Zimbabwe. Finally, policy initiatives should prioritize integrating mental health services into HIV care frameworks and promote collaboration with local organizations and health authorities. This can ensure that policies are sustained overtime. By establishing comprehensive support systems, policymakers can effectively address the challenges encountered by AYALWHIV, prevent child maltreatment, and promote access to better mental health services that can improve their overall well-being in Zimbabwe.

### Limitations of the Study

This study has several limitations worth highlighting. This study is a cross-sectional survey design of the 2017 Zimbabwe Violence Against Children Survey (ZVACS), which prevents causal inference and limits the ability to determine the temporal direction between childhood maltreatment and psychological distress. The cross-sectional design also does not allow for examination of changes in mental health outcomes over time. All measures were self-reported, which may introduce recall and social desirability biases and underreporting. Questions related to sexual abuse and HIV status are particularly sensitive, and stigma surrounding both maltreatment and HIV may have reduced disclosure about these incidences. Psychological distress was assessed using Kessler-6 only for the past 30 days, limiting the ability to capture longer-term or fluctuating mental health effects following victimization. Childhood maltreatment was assessed using survey items capturing physical, emotional, and sexual abuse, and neglect which is part of the child maltreatment was not included in the dataset. Because neglect is strongly associated with long-term emotional and developmental consequences, its absence may underestimate the overall burden of adversity for AYALWHIV. The reliance on retrospective reporting may also result in recall errors or misclassification.

In addition, the survey did not capture the frequency, severity, chronicity, or age of onset of maltreatment, limiting assessment of cumulative or dose–response effects. Contextual details, such as perpetrator characteristics or duration of exposure, were also not fully examined this may influence the outcome if some of the potential indicators are not accounted for. The survey was household-based and excluded youth living in institutional settings or outside family care. As a result, the findings may not represent AYALWHIV who are homeless, incarcerated, hospitalized, or otherwise institutionalizedgroups that may experience increased vulnerability to maltreatment and psychological distress. Although the broader survey was nationally representative consistent of 8,715, we used only the subsample of participants living with HIV which was relatively small (*n* = 249), limiting statistical power for subgroup analyses and constraining external validity or generalization. Other limitations inherent to ZVACS include reliance on standardized survey items, limited clinical mental health assessment beyond screening tools, and lack of longitudinal data (e.g., timing of HIV diagnosis, treatment adherence, or sequencing of maltreatment exposure and distress). Despite these insurmountable limitations, the current study provides important evidence that cumulative child maltreatment exposure is significantly associated with increased risk of developing psychological distress among AYALWHIV, thereby contributing valuable context-specific insights to the literature in Zimbabwe.

## Conclusion

This study highlights that experiencing child maltreatment, particularly sexual and emotional abuse, increases the risk of psychological distress among AYALWHIV. Preventive measures and trauma-informed interventions, including anti-stigma education, mental health support, and community engagement prevention mechanism, are essential for reducing psychological distress associated with child maltreatment among AYALWHIV. Collaborative efforts with local organizations and policy initiatives that can integrate mental health services into HIV care frameworks are crucial for creating comprehensive support systems. These approaches can help improve overall well-being among AYALWHIV living in low-resource settings in Zimbabwe.

## Data Availability

The data used for study will be available upon request from the corresponding author.
